# Seeing the bigger picture: endogenous opioids mediate attentional broadening after reward receipt

**DOI:** 10.1017/S0033291725101815

**Published:** 2025-09-26

**Authors:** Henk van Steenbergen, Philip Gable, Harry Fagan, Laura Molteni, Algirdas Midveris, Nathan Huneke

**Affiliations:** 1 Cognitive Psychology Unit, Institute of Psychology, Leiden University, Leiden, The Netherlands; 2 Leiden Institute for Brain and Cognition, Leiden, The Netherlands; 3 Department of Psychological & Brain Sciences, University of Delaware, Newark, DE, USA; 4 Clinical and Experimental Sciences, Faculty of Medicine, University of Southampton, Southampton, UK; 5 Southern Health National Health Service Foundation Trust, Hampshire, UK

**Keywords:** approach motivation, attentional breadth, mu-opioid receptor, opioids, positive affect

## Abstract

**Background:**

Contemporary society faces significant challenges that can lead to stress-induced tunnel vision. Positive affect can counteract these effects by expanding humans’ attentional scope, potentially promoting resilience and creativity. This preregistered triple-blind study investigated the role of endogenous opioids in mediating attentional broadening following reward receipt.

**Methods:**

Using a placebo-controlled crossover design, 40 volunteers underwent two sessions separated by at least 1 week, receiving either 50 mg of naltrexone or a placebo. Participants completed a Navon letters task designed to contrast the effects of reward receipt versus reward anticipation on attentional scope.

**Results:**

As predicted, our results show that the attentional broadening observed after reward receipt under placebo was eliminated when opioid receptors were blocked. Naltrexone did not result in blunted reward anticipation effects on task performance or attentional narrowing.

**Conclusions:**

This study highlights the role of endogenous opioids in attentional breadth and their potential for cognitive flexibility and resilience through natural positive experiences, with potential implications for mental health and stress management.

## Introduction

Contemporary society faces multiple challenges, from increasing polarization to the looming climate crisis, all of which are putting substantial pressure on individuals. Stress can lead to tunnel vision, potentially impeding the ability to think creatively and find effective solutions to address the root causes of stress (Chajut & Algom, 2003; Easterbrook, 1959; Garland et al., 2010). Research has shown that experiencing positive emotions can help individuals maintain a broader perspective (Dreisbach & Goschke, 2004; Fredrickson, 2001; Isen, 1990), suggesting that the human brain is equipped with a natural neurochemical mechanism that supports broadened attention in response to positive feelings. This mechanism may also play a role in developing a wider range of coping strategies to build resilience in the face of adversity (Garland et al., 2010; Hanif et al., 2012; Whitmer & Gotlib, 2013). However, the specific neurochemical processes linking positive emotions and an expanded attentional scope remain largely unknown.

In this study, we focused on the role of endogenous opioids. These peptides have a broad impact on the brain and play a modulating role in pleasurable experiences (Kringelbach & Berridge, 2009). Studies involving both animals and humans using pharmacological approaches have shown that their effects on mu-opioid receptors partially mediate the pleasurable response to rewards across various modalities and domains (Laurent, Morse, & Balleine, 2015; Meier, Eikemo, & Leknes, 2021; Nummenmaa & Tuominen, 2018). Unlike other reward systems such as the dopamine system, which predominantly mediates reward-seeking and motivation rather than reward enjoyment (Barbano & Cador, 2007; Berridge, 2007; Kringelbach & Berridge, 2009; Webber, Lopez-Gamundi, Stamatovich, de Wit, & Wardle, 2021), mu-opioids play a more global role, as they modulate reward liking as well as reward-related motivational processes (Meier et al., 2021). Moreover, mu-opioids also aid in relieving pain and stress (Leknes & Tracey, 2008; Valentino & Van Bockstaele, 2015). Emerging research shows that other opioid receptor types also play a modulating role in reward and affective states (Darcq & Kieffer, 2018; Lutz & Kieffer, 2013; Meier et al., 2021; Nummenmaa & Tuominen, 2018). However, unlike the extensive research on dopamine and other neuromodulators, the study of the impact of endogenous opioids on cognitive processes, particularly their modulation by affective states, is still in its early stages (Chiew & Braver, 2014; Van Steenbergen, Eikemo, & Leknes, 2019).

Previous studies have shown that positive emotional states with low approach motivation can broaden attention (Gable & Harmon-Jones, 2011; Lacey, Wilhelm, & Gable, 2021; Vanlessen, De Raedt, Koster, & Pourtois, 2016). For example, receiving a reward after completing a task has been found to expand attention to global visual features in a subsequent task. Conversely, when participants anticipate a reward but have not yet completed the task required to receive it, attention tends to be focused on details. This phenomenon has been consistently observed in several behavioral experiments using the Navon task, which features small shapes arranged in the form of a larger shape (Gable & Harmon-Jones, 2011; Sadowski & Fennis, 2020). The distinct attentional effects of post-goal and pre-goal rewards have been suggested to be linked to partially separable PLAY/SEEKING (Burgdorf & Panksepp, 2006) or liking/wanting (Berridge, 2007) neurochemical systems in the brain (Gable & Harmon-Jones, 2008). However, this connection has not been empirically confirmed yet.

We combined the pre-/post-goal reward attentional scope task developed by Gable and Harmon-Jones (2011) with a pharmacological intervention using the non-specific opioid blocker naltrexone and a placebo in a preregistered triple-blind randomized within-subject crossover design. We used a dose that blocks the majority (>90%) of mu-opioid receptors, along with a partial blockage of the delta and kappa receptors in the brain (Weerts et al., 2013), allowing us to directly test a mediating role of the endogenous opioid system in reward-induced attentional scope modulation. Our main hypothesis was that if opioid release is involved in the link between reward receipt and attentional scope, we would observe that attention broadening after reward receipt in the placebo condition would not occur when opioid receptors are blocked (preregistered primary hypothesis H1). Additionally, given that mu-opioids can also influence reward wanting (Meier et al., 2021), we hypothesized that naltrexone may blunt reward anticipation effects on task performance and attentional narrowing as well (preregistered secondary hypothesis H2). We also expected that the drug effects were not associated with mood changes assessed before the task (preregistered secondary hypothesis H3).

## Methods

### Preregistration

Methods, hypotheses, and analyses were preregistered (https://osf.io/tbv8d) and deviations (all minor) from preregistration are described in the text and fully reported in Supplementary Table S1.

### Participants

Forty healthy volunteers aged between 18 and 55 years (*M* = 24.4 years; 16 males) were recruited to attend Southampton General Hospital for a multi-session study focused on threat processing and neuroimaging (results to be published elsewhere). Exclusion criteria included: any history of, or current, psychiatric illness, any current medical illness, use of medication in the last 8 weeks, regular use of illicit substances, and any contraindication to naltrexone. A complete list of exclusion criteria is listed in the supplementary material (Supplementary Table S2).

One participant dropped out after randomization but before data collection, and data from another participant were unavailable due to a technical issue. Upon screening the remaining data, participants with extreme outlier error rates per session (naltrexone versus placebo) were excluded from the analysis (three participants) using the 3x IQR criterion. We deviated from our preregistration plan by not applying the same criterion for omission rates, as this would have eliminated half of our sample. Note that the omission rate was small, ranging from 0% to 6%.

The sample size was optimized for the fMRI part of this study as this would provide power of 0.80 to detect a moderate effect size of 0.5–0.6 between conditions with an alpha of 0.05 using a voxelwise correction of family-wise error rate. Estimating the effect size of naltrexone on reward-related modulations of attentional scope is challenging given the novelty of the approach used here. Therefore, we used the average effect size in psychology as a reference, which is typically small-to-medium for within-subject effects (Cohen’s *d* ~ .41). With our directional preregistered hypotheses, we conducted one-sided tests for planned comparisons (Hales, 2024). With 35 participants included in the analysis, we have 77% power to detect effects of this magnitude (calculated using GPower 3.1.97).

### Design

We used a randomized within-subject crossover design with two sessions. Approximately 100 minutes after taking the drug, participants completed questionnaires including the PANAS and an adapted Navon task with a monetary incentive during both sessions (details below). Participants were randomly assigned to receive either a 50-mg naltrexone tablet or a placebo in matching capsules. Naltrexone is a non-specific opioid antagonist that primarily targets mu-opioid receptors. Sessions were scheduled at least 7 days apart to reduce the impact of carry-over effects, although recent work suggests a minimum interval of 15 days to ensure complete elimination (Trøstheim, Eikemo, Haaker, Frost, & Leknes, 2023). Medication was administered by an independent investigator to maintain blinding of treatment assignment for both the participant and investigator. Following each session, participants were debriefed and had their blood pressure and heart rate checked to ensure they were ready to leave.

### Pre-/post-goal reward attentional scope task

**Figure 1.**
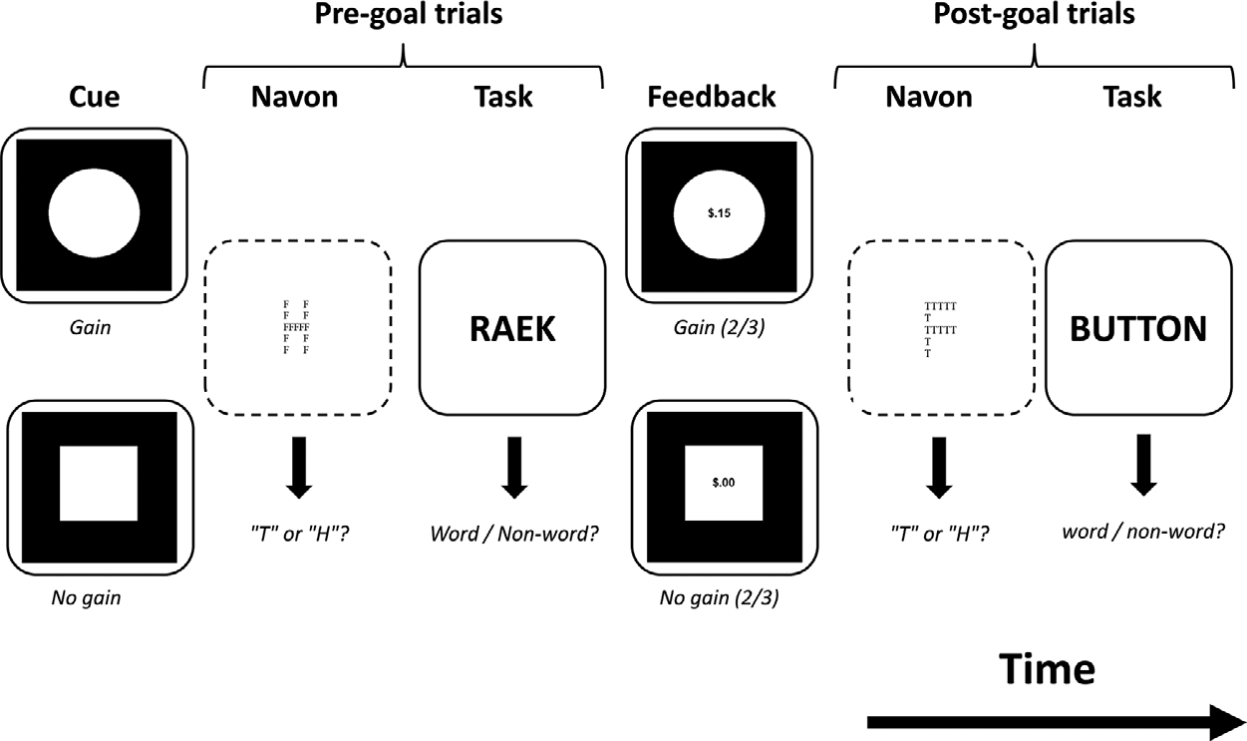
Example trials of the pre-/post-goal reward attentional scope task showing expected feedback following a correct response to the lexical decision task. The Navon stimuli (dashed) were presented in a subset of trials to probe attentional scope. In this task, reward expectation has been observed to narrow attention in the pre-goal Navon stimulus, whereas reward receipt has been observed to broaden attention in the post-goal Navon task (Gable & Harmon-Jones, 2011).

The task presented was based on Experiment 2 from Gable and Harmon-Jones (2011), using the same stimuli and software as the original study. Participants engaged in a modified monetary incentive delay task, where a cue before the main task prompted them to determine if a given stimulus was a word or nonword, followed by feedback. As shown in Figure 1, attentional breadth was assessed both before (pre-goal) and after (post-goal) the task by including a Navon task after the cue and/or feedback in a subset of the 96 total trials. After receiving a visual cue indicating whether they could gain money in that trial or not (48 trials for both gain and no-gain cues), participants occasionally responded to an undirected Navon stimulus in which participants had to detect the letter T or H (pre-goal Navon task; 32 trials in total). This target was present either in the global (e.g., a large T that consists of small Fs) or the local feature of the stimulus (e.g., a large F that consists of small Ts). After that, participants were presented with a (non)word and had to indicate whether the word presented was a real word or a nonword (lexical decision task; 96 trials). Participants then received feedback indicating whether money was gained (i.e., showing the text ‘$.15’ or ‘$.00’). In two-thirds of the trials, they received gain feedback if the preceding cue indicated gain, and in two-thirds of the trials, they received no-gain feedback if the preceding cue indicated no gain. Opposite (unexpected) feedback was given during the other one-third of trials. As preregistered, the analyses focus only on the post-goal trials with expected feedback. If participants responded incorrectly to the lexical decision task, they were always given post-goal no-gain feedback. As in the original paper, these trials were excluded from analyses (see Supplementary Table S1). Following the feedback, participants occasionally saw another Navon stimulus (64 trials in total). A trial concluded with another lexical decision task that was presented to balance the design (96 trials in total), though not included in the analyses. Cue and feedback stimuli were displayed for 2000 ms, and participants were required to respond with either their left or right hand during the Navon and lexical-decision tasks within a 4000 ms response window.

Participants were incentivized with a bonus that could be exchanged for four Snickers bars upon completion of the study session. Although instructions emphasized that the bonus would be based on performance, all participants received the same reward at the end of the session. We did not include ratings on the perceived pleasantness of the cues/rewards in this study.

### Analyses

As preregistered, all behavioral analyses were run separately for the following three subsets of trials: expected post-goal reward Navon trials, pre-goal reward Navon trials, and pre-goal reward lexical decision task trials. Data were unblinded after the main analyses were run but before robustness checks (see below) were performed. During preprocessing, we visually inspected the distribution of the single-trial reaction time (RT) data for each combination of condition and participant separately. The distribution was not clearly skewed to the right, so no log transformation was applied. RT analyses included only correct responses. RTs deviating more than two standard deviations from each condition-specific mean for each participant separately were removed (1.75%, 2.14%, and 5.19% respectively, for the three subsets of trials indicated above).

In addition, the aggregated data (means per cell per participant) were screened for potential participant outliers using box plots. As we described in the preregistration, repeated measures ANOVAs are quite robust for outliers (Blanca Mena, Arnau Gras, García de Castro, Alarcón Postigo, & Bono Cabré, 2023), so we preregistered to include potential outliers in the data. However, inspection of the distribution of the residuals revealed that normality was seriously violated for most of these analyses (see Supplementary Figure S1). We, therefore, performed robustness checks by repeating our analyses using transformations that considerably improved the normality of the residuals, using a reciprocal transformation for RT (Response Speed = 1000/RT) and a square root transformation for error rate. Results of the untransformed analyses are presented in the Supplement. Aiming for robust findings, we only report effects from the ANOVAs when they are significant for both the untransformed and transformed variables. Supplementary Figure S2 presents the results based on the untransformed RT findings.

For the Navon pre-goal and post-goal trial analyses, we used a 2 (Reward: gain vs. no-gain) × 2 (Navon Type: local vs. global) × 2 (Drug: naltrexone vs. placebo) × 2 (Order: naltrexone in first session, placebo in second session vs. placebo in first session, naltrexone in second session) repeated measures ANOVA. All factors are within-subject except Order. For the analysis of the lexical decision task, the factor Navon Type was removed, and the factor Word Type (word vs. non-word) was added. Descriptives are presented in Supplementary Tables S3–S5. All ANOVA results are presented in Supplementary Tables S6–S11.

**Figure 2.**
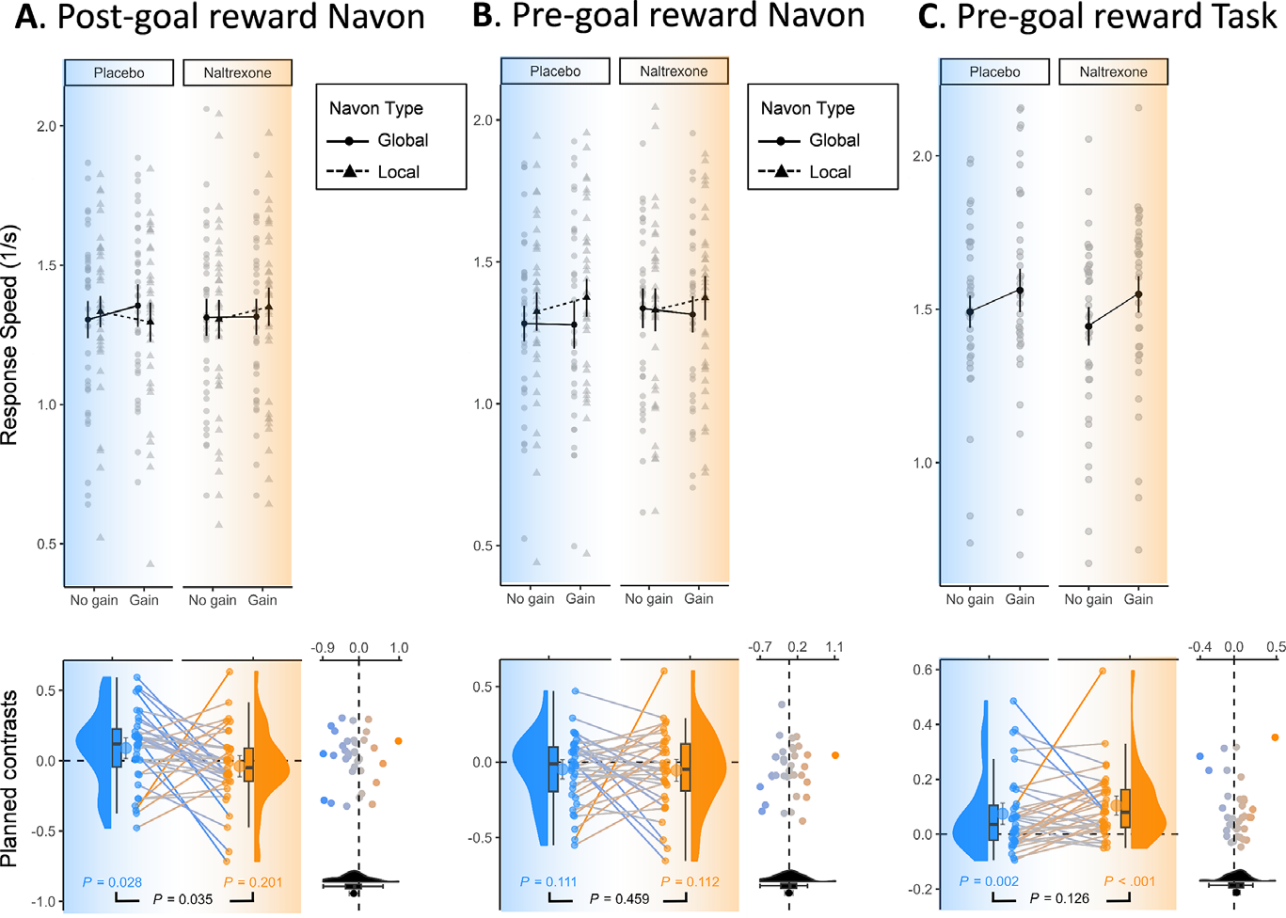
Opioid blockade (Naltrexone condition; right side) relative to placebo (Control condition; left side) selectively eliminated attentional broadening after reward receipt (A), but did not change attentional narrowing after reward anticipation (B) or reward-related performance speed on the lexical decision task (C). Upper panels show the mean and 95% within-subject confidence intervals, and dots indicate data from each participant. Lower panels show the values of the planned contrast of interest to test the interaction between Reward and Navon Type (A and B; difference of difference scores) and the main effect of Reward (C; difference score) against zero (dotted horizontal line) for the placebo (left side) and naltrexone (right side) condition separately. In A and B, positive planned-contrast values indicate more attentional broadening (quicker responses during global than local trials) in the gain than the no-gain condition, that is, (GainGlobal – GainLocal) – (NoGainGlobal – NoGainLocal). In C, positive planned-contrast values indicate quicker responses in the gain than the no-gain condition, that is, (Gain – NoGain). The paired difference between these contrasts, reflecting the drug effect on these planned-contrast values (i.e., the slope of the lines connecting the dots), is plotted in the scatterplot (x-axis) as a function of the average of both values (y-axis) and was also tested against zero (dotted vertical line). The error bars show the mean and 90% within-subject confidence intervals, and reported p-values are one-tailed and use t-tests against zero that ignore all other factors of the design.

All preregistered predicted interactions were tested using planned comparisons (i.e., transforming the omnibus F-test into a t-test) and planned contrasts (difference of difference scores tested against zero, i.e., pair-wise comparisons ignoring other factors in the design, see caption in Figure 2). We used one-tailed tests for these comparisons and contrasts, as they control for the same rate of false positives as two-tailed tests when preregistered, while being more powerful to detect true effects (Hales, 2024). For consistency, one-tailed tests and 90% confidence intervals are always reported for our planned comparisons, even if the pattern was observed to be (numerically) opposite in direction. For all other effects, we report two-tailed tests and 95% confidence intervals. All behavioral analyses were run in RStudio (version 2023.09.0 build 463) using R (version 4.3.0). The plots in the upper panels of Figure 2 were created using the afex_plot function from the afex package version 1.3–1 (Singmann, Bolker, Westfall, Aust, & Ben-Shachar, 2024). The plots in the lower panels of Figure 2 were created based on customized code derived from the raincloud plot package (Allen et al., 2019).

Concerning subjective effects of the drug, we expected to find evidence for the null hypothesis, that is, that the drug does not have a main effect on self-reported mood states. To test this, we submitted the Positive Affect and Negative Affect scores of the PANAS questionnaire to a Bayesian RM ANOVA with the factors drug (within-subject) and order (between-subject) using the default priors available in JASP software version 0.18.3 (JASP Team, 2024).

## Results

### Post-goal reward broadens attention and this effect is blocked by naltrexone

Descriptive statistics are presented in Supplementary Table S3. Consistent with our preregistered main hypothesis, we observed the expected three-way interaction between Reward, Navon Type, and Drug in response speed, *t*(33) = −1.86, *p_one-tailed_* = 0.036, *η^2^* = .003; planned-contrast value *M [CI] =* −0.13 [−0.24,-0.02], *p_one-tailed_* = .035, Hedges’ *g* = .309). As Figure 2A shows, we replicated the earlier reported broadening of attention after post-goal reward during placebo in the two-way interaction between Reward and Navon Type (planned-contrast value *M [CI] =* 0.09 [0.01,0.16], *p_one-tailed_* = .028, Hedges’ *g* = 0.325). That is, after gain (relative to no-gain), participants were faster to detect the global Navon letter than the local Navon letter. Critically, this effect was no longer observed when the influence of endogenous opioids was pharmacologically blocked with naltrexone (planned-contrast value *M [CI] =* −0.04 [−0.11,0.04], *p_one-tailed_* = .201, Hedges’ *g* = −0.140). In addition, a full cross-over Drug × Order effect was observed, *F*(1, 33) = 38.96, *p* < .001, *η^2^* = .103, indicating that participants became faster during the second session. No significant effects were observed in the error rate.

### Pre-goal reward narrows attention

Descriptive statistics are presented in Supplementary Table S4. Replicating earlier work, in the pre-goal Navon trials, we observed a Reward × Navon Type effect in response speed, showing that reward anticipation narrows attention, *F*(1, 33) = 4.37, *p* = .044, *η^2^* = .002. That is, after a gain cue (relative to a no-gain cue), participants were faster to detect the local Navon letter than the global Navon letter. However, we did not confirm the secondary hypothesis that naltrexone blocks this effect; the effect was numerically in the opposite direction, *t*(33) = 0.17, *p_one-tailed_* = 0.4325, *η^2^* <.001, planned-contrast value *M [CI] =* −0.03 [−0.08,0.02], Hedges’ *g* = −0.156, *p_one-tailed_* = .175. As Figure 2B shows, following a gain cue (relative to a no-gain cue), participants were numerically faster to detect a local Navon letter than a global Navon letter, both in the Placebo condition and in the Naltrexone condition, although this effect was weak and was not significant in the separate planned contrasts split for Drug. In addition, a full cross-over Drug × Order effect was observed, *F*(1, 33) = 27.50, *p* < .001, *η^2^* = .097, indicating that participants became faster during the second session. No significant effects were observed in the error rate.

### Pre-goal reward speeds up lexical decision performance

Descriptive statistics are presented in Supplementary Table S5. Replicating earlier work, in the pre-goal lexical decision task trials we observed a main effect of Reward, *F*(1, 33) = 21.71, *p* < .001, *η^2^* = .021, and a main effect of Word Type, *F*(1, 33) = 238.50, *p* < .001, *η^2^* = .222, indicating that participant performed faster when a reward was at stake, and responded slower to non-words relative to words (see Figure 2C). In addition, a full cross-over Drug × Order effect was observed indicating that participants became faster during the second session, *F*(1, 33) = 38.03, *p* < .001, *η^2^* = .105. However, we could not confirm our secondary hypothesis that naltrexone blocked the reward effect on lexical decision task speed, and the effect was numerically in the opposite direction *t*(33) = 1.34, *p_one-tailed_* = 0.095, *η^2^* <.001, planned-contrast value *M [CI] =* 0.03 [−0.01,0.07], *p_one-tailed_* = .126, Hedges’ *g* = 0.192. Faster responses to the gain versus no gain cue were reliably observed in the Placebo condition, planned-contrast value *M [CI] =* 0.07 [0.04,0.11], *p_one-tailed_* = 0.002, Hedges’ *g* = 0.52, and in the Naltrexone condition, *M [CI] =* 0.10 [0.07,0.14], *p_one-tailed_* < .001, Hedges’ *g* = 0.812.

Analyses on error rate showed that participants made more errors when the reward was at stake, *F*(1, 33) = 7.59, *p* = .009, *η^2^* = .021. Combined with the reward-induced increase in speed, this suggests that reward prospect increased impulsiveness in the lexical decision task. In addition, participants made more errors with non-words relative to words, *F*(1, 33) = 23.87, *p* < .001, *η^2^* = .153. No other effects were significant.

### No credible drug effects on mood

State Positive Affect and Negative Affect measured with the PANAS before the Navon task did not reveal noticeable drug effects (*M* = 22.7 and *M* = 10.9 for placebo; *M* = 22.5 and *M* = 11.4 for naltrexone). There was moderate evidence against including the factor Drug (*BF_excl_* = 1/0.246 = 4.06) for Positive Affect, whereas evidence was inconclusive for Negative Affect (*BF_incl_* = 0.631).

## Discussion

In our preregistered study, we found that the typical attentional broadening observed after reward receipt (Gable & Harmon-Jones, 2011) was no longer present when we pharmacologically blocked opioid receptors using naltrexone. These findings provide initial support for our primary hypothesis that endogenous opioids play a mediating role in the connection between attentional broadening and post-goal positive affect. Our results are the first to hint at a neurochemical mechanism that underlies attentional broadening that has been attributed to distinct positive affective states for decades (Dreisbach & Goschke, 2004; Fredrickson, 2001; Isen, 1990). These effects occurred without any discernible impact of the drug on pre-task mood state.

Our novel findings extend the literature on the opioid modulation of social rewards and attentional processes. For example, opioid agonists are known to increase the visual exploration of faces in humans, whereas antagonists reduce this (Chelnokova et al., 2016). Moreover, in rodents, exploratory (File, 1980) and playful behavior has been linked to the mu-opioid system (Trezza, Baarendse, & Vanderschuren, 2010), and the latter effects occur at the post-goal rather than pre-goal stage in some work (Normansell & Panksepp, 1990), but see also (Achterberg, van Swieten, Houwing, Trezza, & Vanderschuren, 2019). We note that the drug effects on attention in our task were specific to the post-goal reward condition; that is, naltrexone did not induce a global reduction of attentional scope across task conditions. This finding aligns with human studies that also do not show clear opioid-driven global attentional effects and instead suggest that opioids help to fine-tune changes in cognitive processes in an affective context (Van Steenbergen et al., 2019).

At the same time, we did not observe that naltrexone blunted the effects of reward anticipation on task performance or attentional narrowing. This finding does not align with evidence showing that mu-opioids modulate both reward reception and reward anticipation (Meier et al., 2021). Notably, studies that compared reward anticipation and receipt in the same design have yielded mixed findings, with some work observing opioid modulation during both phases (Korb et al., 2020), whereas others observed effects only during reward receipt (Buchel, Miedl, & Sprenger, 2018; Massaccesi et al., 2024). Bearing the usual caveats in mind when interpreting null findings, one possibility may be that other neurochemicals, such as catecholamines, particularly dopamine (Chiew & Braver, 2014), play a more prominent role in reward anticipation and attentional narrowing than opioids. Indeed, reward-related attentional focus has been linked to dopamine in previous work (Aarts et al., 2014; Anderson et al., 2016; Westbrook & Braver, 2016), although the role of dopamine in reward processing is complex (Webber et al., 2021).

More broadly, our findings align with recent studies that have begun to highlight the complexity of positive affect, including its relationship to cognitive functions and neurochemistry. From a dimensional perspective, positive affect is a concept that can only be properly captured when affective space is conceptualized as a hypercube, i.e., requiring more than two or three dimensions (Fontaine, Scherer, Roesch, & Ellsworth, 2007). Distinct affective states may be conceived as points projected onto this affective space and serve specific functions rooted in evolutionary history, probably involving overlapping and only partially separable neurochemical systems (Burgdorf & Panksepp, 2006; Nummenmaa & Tuominen, 2018; Shiota et al., 2017). With regard to the affective modulation of cognition, the level of motivational intensity, or the impetus to act, might be a key dimension in this space that cannot be reduced to valence and/or arousal (Harmon-Jones, Price, & Gable, 2012; Lacey et al., 2021; Paul, Pourtois, van Steenbergen, Gable, & Dreisbach, 2021). Specifically, low-approach positive affect has been shown to not only facilitate a broadened attentional scope, as replicated here, but also to facilitate behavioral flexibility (Liu & Wang, 2014). It has been proposed that these cognitive effects are associated with discrete emotional states such as contentment, serenity, and amusement (Lacey et al., 2021). Our findings hint at the possibility that endogenous opioids play a role in the affective modulation of cognition for these states, that is, affective states that are low in approach motivation, although this obviously does not preclude a role for other neurochemical modulators. High approach motivation affect, on the other hand, is expected to yield opposite cognitive consequences, including focused attention and goal maintenance (Harmon-Jones et al., 2012; Lacey et al., 2021; Paul et al., 2021). While neurochemical systems are complex and can have non-linear and interactive effects, it could be speculated that neurochemical systems other than opioids, such as the dopamine system, play a more prominent role in those high approach motivation states (Paul et al., 2021; Webber et al., 2021).

A few limitations warrant further consideration. First, our pharmacological study used a non-specific opioid blocker. Although our findings are consistent with earlier work suggesting the involvement of the mu-opioid system in reward, we cannot rule out the effects of other opioid receptor types that are also partially blocked by naltrexone, such as kappa and, to a lesser extent, delta receptors (Meier et al., 2021). Nevertheless, the observed post-goal reward modulation in our study is largely consistent with studies revealing bidirectional effects on reward processing when combining a similar antagonist with an agonist that primarily binds to mu-opioid receptors (Meier et al., 2021). Second, we did not assess the subjective pleasantness of the rewards. Earlier work has shown that opioid modulation is most pronounced for hedonic ratings of high-value, but not low-value, social, erotic, monetary, and food rewards (Buchel et al., 2018; Chelnokova et al., 2014; Eikemo et al., 2016; Petrovic et al., 2008), whereas null effects on liking have also been reported (Korb et al., 2020; Kut et al., 2011; Massaccesi et al., 2024). Given the low-value rewards employed in our task, it could be that endogenous opioids modulated the *link* between reward receipt and attentional breadth, rather than directly modulating the reward pleasantness itself. Although future research is needed to test this account and its neural mechanisms, such a more complex mechanism also seems consistent with the notion that mu-opioid receptors fine-tune rather than fully mediate hedonic states (Eikemo, Løseth, & Leknes, 2021).

To conclude, we demonstrated for the first time that endogenous opioids modulate attentional broadening following the receipt of a reward but do not reliably mediate attentional narrowing during reward anticipation. This finding suggests that endogenous opioids modulate affect-induced tuning of cognition, which could also beneficially impact mental health (Garland et al., 2010; Hanif et al., 2012; Whitmer & Gotlib, 2013). Past research has already shown that engaging in activities such as listening to pleasant music can activate the opioid system (Nummenmaa & Tuominen, 2018), potentially reducing the reliance on opioid medications for pain management in medical contexts (Chai et al., 2017). In a world filled with increasing stress and alarming rates of opioid misuse, our findings underscore that endogenous opioids are a natural resource that humans possess, which can help expand our mental outlook in response to pleasant experiences (Alexander et al., 2021). An interesting avenue for future work is to identify whether the altered cognitive tuning induced by endogenous opioids may, in turn, also contribute to our capacity for flexible behavior and foster resilience (Garland et al., 2010; Hanif et al., 2012; Whitmer & Gotlib, 2013).

## Supporting information

van Steenbergen et al. supplementary material van Steenbergen et al. supplementary material

## Supplementary material

The supplementary material for this article can be found at http://doi.org/10.1017/S0033291725101815.

## Significance

To effectively manage daily challenges and stress, individuals must avoid tunnel vision and rigid thinking. For decades, researchers have proposed that experiencing positive emotions can help expand one’s attentional breadth and improve cognitive flexibility. However, the quest for the neurochemical mechanisms underlying this process has yielded limited insights. Our research indicates that endogenous opioids, known to be activated by pleasurable experiences, play a substantial role in attentional broadening following reward receipt, paving the way for a new research program on the role of endogenous opioids in cognitive flexibility and resilience. In an era of concerning levels of opioid misuse, our findings also underscore the effects of natural brain opioids as an antidote to stress and its cognitive consequences.

## Research transparency statement

Conflicts of interest: All authors declare no conflicts of interest. Funding: HvS was supported by the interdisciplinary research program ‘Social Resilience and Security’ of Leiden University, and HAF was supported in this work by the University of Southampton National Institute for Health Research Academic Foundation Programme. Artificial intelligence: During the preparation of this work, the authors used ChatGPT to improve language and readability. After using this tool service, the authors reviewed and edited the content as needed, and they take full responsibility for the content of the publication. Ethics: This research received approval from the Ethics Psychology Committee of the University of Southampton (ERGO ID 53540). Computational reproducibility: all analyses can be reproduced in R using the scripts publicly available.

Preregistration: The hypotheses, methods, and analyses were preregistered (https://osf.io/tbv8d) on 2021-05-08, while data collection was ongoing (data collection of seven participants was completed). There were minor deviations from the preregistration (for details, see Supplementary Table S1). Materials: The Navon task is publicly available on OSF (https://osf.io/kufts/). Data: All data and analysis scripts are publicly available (https://osf.io/kufts/).
