# Supplementary material for: Seeing the bigger picture: endogenous opioids mediate attentional broadening after reward receipt
Source: Psychol Med. 2025 Sep 26;55:e283. doi: 10.1017/S0033291725101815 (PMC12527506; doi:10.1017/S0033291725101815)
Supplement: van Steenbergen et al. supplementary material [file S0033291725101815sup001.pdf]

Table S1 Preregistration deviations

#	Details		Original wording	Deviation description	To what extent is this a deviation from the preregistered plan?	Judgment of impact
1	Type<br>Reason<br>Timing	Analyses<br>New knowledge<br>After data access	Participants with average error rates or omission rates per session (naltrexone versus placebo) that are extreme outliers (3 IQR criterion in SPSS) will be excluded from the data.	We did not consider this criterion when analyzing omission rates, as participants rarely failed to respond (only 1% to 6% of trials). The vast majority of participants did not omit any responses, resulting in Q1 and Q3 values of 0%, so invalidating the 3*IQR (= 3 * 0) criterion due to the lack of variance. Applying this criterion anyway would exclude half of our sample (17 additional participants out of the 34 participants we analyze now).	Minor	Excluding these participants would dramatically lower the statistical power and none of the participants had numerically extremely high omission rates anyway. Therefore, we have decided not to analyze the data without these participants.
2	Type<br>Reason<br>Timing	Analyses<br>New knowledge<br>After results known	In addition, the aggregated data (means per cell per participant) will be screened for potential outliers using boxplots. If the distribution of average error rates is skewed, we will apply a square-root transformation.<br>Repeated measures ANOVAs	Inspection of the distribution of the residuals revealed that normality was seriously violated for most of our analyses on nontransformed data (see Figure S1). We therefore repeated our analyses using transformations that	Minor	Aiming at robust findings, we only report effects from the ANOVAs when they are significant for both the untransformed and transformed variables.

			are quite robust for outliers so we will include potential outliers in the data and report greenhouse-geisser corrected statistics in case the assumption of sphericity is violated.	considerably improved the normality of the residuals, using a reciprocal transformation for RT (Response Speed = 1000 / RT) and a square root analyses for error rate. Results of the untransformed analyses are presented in the Supplement.<br>Note also that greenhouse-geisser correction cannot be applied because this can only be calculated when the number of levels of predictors in the ANOVA exceed two.		
3	Type<br>Reason<br>Timing	Analyses<br>Typo/Error<br>After data access	[Not described in pre-registration]. Gable & Harmon-Jones (2011, p 1360) state: "Also, if participants responded incorrectly to the flankers [= lexical decision-task in Experiment 2]<br>task, they were given postgoal no-gain feedback, and the trial was<br>excluded from analyses"	We inadvertently did not specify this explicitly in the preregistration: For the Navon trial analyses we excluded trials in which participants responded incorrectly to the lexical decision task (which never resulted in gain, see above).	Minor	We aim to replicate the original analytic approach by Gable & Harmon-Jones (2011) so deviating from it will lead to incorrect inferences: Given that errors never lead to a gain, they should not be analyzed as gain trials. Neither should errors for no-gain cue trials be included because they can introduce post-error adjustments in behavior, biasing the results and invalidating comparisons between conditions.
4	Type	Analyses	Statistical analyses will be	The pre-registered main	Minor	Minimal impact given

Reason	New knowledge	performed prior to unblinding.	analyses were indeed performed prior to unblinding. However, the robustness checks were performed after unblinding.		that we only report effects from the ANOVAs when they are significant for both the untransformed and transformed variables.
Timing	After results known				

*Note that the risk of bias incurred by a deviation is often unknown. Such uncertainty should be acknowledged and factored into any relevant claims.

Table S2 Summary of exclusion criteria

Condition	Reason	Assessment	Eligibility
Age	Under 18 or over 55	Interview	Not eligible
Caffeine	>8 cups per day	Interview	Not eligible
Alcohol	>21 per week<br>History of alcohol dependence/abuse	Interview	Any = not eligible
	MINI questions concerning drinking behaviours (2/2)<br>Positive breath test	Interview	Not eligible
Smoking	> 6 per day	Interview	Not eligible
Cardiovascular	Hypertension<br>Cardiovascular disease	Interview	Any = not eligible
BMI	Less than 18, more than 28	Interview – reassessed in the inhalation day screening session	Not eligible
Pregnancy	Pregnant<br>Breast feeding	Interview and inhalation day screening session	Not eligible
Handedness	Left or mixed hander	Edinburgh handedness inventory	Not eligible
MRI Safety Screening	Unsafe for scanning according to screening questionnaire	MRI screening questionnaire	Not eligible
Depression	History or current diagnosis of bipolar/MDD	Interview	Not eligible
	2/3 of the depression questions on the MINI	Interview	Not eligible
Mania	History of mania	Interview	Not eligible

	2/3 questions on the MINI	Interview	Not eligible
Panic	History or diagnosis of panic disorder	Interview	Not eligible
	History of panic attacks/hyperventilation attacks		
	2/2 of the anxiety questions on the MINI	Interview	
GAD	History or diagnosis of GAD	Interview	Not eligible
	2/2 of the GAD MINI questions	Interview	Not eligible
PTSD	History or diagnosis of PTSD	Interview	Not eligible
	Questions on the MINI	Interview	Not eligible
Addiction	Indication of drug abuse	Interview	Not eligible
	Drug use in the last 8 weeks		
	MINI questions for alcohol abuse 2/2		
	Addiction MINI section (individually assessed based on drug use – exceeding occasional (more than twice) use)		
Family history	Family history of panic disorder/panic attacks	Interview and reassessed in inhalation day screening session	Not eligible
Heart and lung disease	Includes diagnosis of asthma (childhood asthma is not included)	Interview	Not eligible
Thyroid disease	diagnosis	Interview	Not eligible
Diabetes	Treatment or diagnosis of diabetes	Interview	Not eligible
Migraines	History of migraines requiring treatment	Interview	Not eligible

Medication use	Any medication use in past 8 weeks (apart from paracetamol, aspirin, local treatments, contraceptives)<br>HRT	Interview and reassessed in inhalation day screening session	Not eligible
Drug/food allergies	Severe allergies	Interview	Not eligible
Epilepsy	Diagnosis of epilepsy<br>History or seizures	Interview	Any = not eligible
Participation in another trial	Current participation in a medical trial. Recent completion of a medical trial	Interview	Not eligible
Naltrexone	Known hypersensitivity to naltrexone<br>Diagnosis of opioid dependence<br>Current opioid misuse	Interview, reassessed at each screening session	Not eligible

Table S3. Descriptive statistics post-goal Navon trials

Order	N	Drug	Reward	Type	RT		RS		ErrorRate		ErrorRate_sqrt	
					Mean	SE	Mean	SE	Mean	SE	Mean	SE
Naltrexone-Placebo	20	Placebo	No-gain	global	785.327	50.144	1.352	0.068	0.108	0.022	0.260	0.046
				local	729.883	32.724	1.414	0.053	0.073	0.023	0.168	0.048
			Gain	global	719.796	33.647	1.442	0.060	0.057	0.024	0.127	0.046
				local	732.112	34.302	1.412	0.053	0.051	0.016	0.133	0.042
		Naltrexone	No-gain	global	884.424	47.522	1.185	0.055	0.077	0.033	0.146	0.054
				local	879.621	52.396	1.204	0.061	0.104	0.031	0.211	0.056
			Gain	global	871.049	45.084	1.198	0.053	0.065	0.028	0.135	0.050
				local	854.644	48.785	1.238	0.064	0.078	0.023	0.183	0.048
Placebo-Naltrexone	15	Placebo	No-gain	global	867.435	75.831	1.257	0.089	0.099	0.036	0.207	0.063
				local	881.894	90.519	1.252	0.088	0.091	0.042	0.168	0.067
			Gain	global	837.016	58.613	1.268	0.078	0.108	0.050	0.196	0.071
				local	952.842	112.430	1.180	0.087	0.069	0.025	0.163	0.055
		Naltrexone	No-gain	global	733.060	49.367	1.440	0.084	0.112	0.039	0.222	0.067
				local	792.144	86.681	1.408	0.102	0.089	0.029	0.201	0.059
			Gain	global	737.046	51.445	1.432	0.080	0.037	0.016	0.099	0.044
				local	734.931	67.475	1.463	0.085	0.080	0.021	0.205	0.052

Table S4. Descriptive statistics pre-goal Navon trials

Order	N	Drug	Reward	Type	RT		RS		ErrorRate		ErrorRate_sqrt	
					Mean	SE	Mean	SE	Mean	SE	Mean	SE
Naltrexone-Placebo	20	Placebo	No-gain	global	755.882	37.854	1.379	0.061	0.059	0.025	0.120	0.048
				local	729.738	32.684	1.418	0.057	0.068	0.020	0.164	0.047
			Gain	global	748.951	41.983	1.408	0.070	0.067	0.033	0.124	0.052
				local	708.258	33.336	1.466	0.062	0.028	0.016	0.064	0.035
		Naltrexone	No-gain	global	862.915	50.752	1.218	0.055	0.085	0.040	0.140	0.059
				local	863.986	48.983	1.225	0.064	0.058	0.023	0.129	0.047
			Gain	global	833.727	47.066	1.264	0.063	0.115	0.040	0.207	0.062
				local	823.192	42.078	1.271	0.060	0.057	0.023	0.128	0.046
Placebo-Naltrexone	15	Placebo	No-gain	global	918.686	84.564	1.187	0.083	0.066	0.024	0.160	0.054
				local	918.139	110.891	1.232	0.095	0.068	0.024	0.163	0.054
			Gain	global	960.849	91.564	1.149	0.091	0.117	0.042	0.213	0.071
				local	863.550	97.282	1.282	0.089	0.062	0.018	0.170	0.049
		Naltrexone	No-gain	global	728.251	53.444	1.455	0.082	0.092	0.026	0.218	0.056
				local	756.200	71.292	1.437	0.094	0.054	0.018	0.147	0.048
			Gain	global	788.162	61.971	1.365	0.091	0.144	0.036	0.289	0.066
				local	719.227	54.518	1.474	0.082	0.069	0.029	0.148	0.058

Table S5. Descriptive statistics pre-goal lexical-decision task trials

Order	N	Drug	Reward	Type	RT		RS		ErrorRate		ErrorRate_sqrt	
					Mean	SE	Mean	SE	Mean	SE	Mean	SE
Naltrexone-Placebo	20	Placebo	No-gain	Non-word	697.946	21.211	1.458	0.045	0.102	0.020	0.268	0.040
				Word	587.272	18.616	1.734	0.053	0.072	0.020	0.214	0.037
			Gain	Non-word	666.234	27.166	1.547	0.060	0.136	0.032	0.313	0.045
				Word	556.037	21.570	1.850	0.071	0.065	0.016	0.220	0.030
		Naltrexone	No-gain	Non-word	889.508	70.760	1.210	0.063	0.133	0.027	0.308	0.045
				Word	694.307	38.324	1.512	0.071	0.040	0.013	0.134	0.034
			Gain	Non-word	833.719	69.630	1.292	0.063	0.102	0.020	0.281	0.035
				Word	634.267	32.900	1.640	0.066	0.056	0.009	0.206	0.027
Placebo-Naltrexone	15	Placebo	No-gain	Non-word	895.536	81.533	1.214	0.081	0.119	0.024	0.306	0.042
				Word	677.936	48.962	1.562	0.089	0.021	0.009	0.082	0.032
			Gain	Non-word	870.528	79.783	1.251	0.084	0.152	0.024	0.372	0.031
				Word	672.680	57.694	1.600	0.101	0.046	0.011	0.172	0.035
		Naltrexone	No-gain	Non-word	776.621	75.055	1.386	0.076	0.107	0.022	0.290	0.041
				Word	631.135	43.794	1.671	0.091	0.037	0.007	0.163	0.028
			Gain	Non-word	716.922	52.854	1.467	0.073	0.139	0.030	0.329	0.047
				Word	583.984	38.693	1.798	0.094	0.072	0.018	0.229	0.037

Table S6. Repeated measures ANOVAs RT and RS post-goal Navon trials

	RT					RS				
Effect	df	MSE	F	η²	p	df	MSE	F	η²	p
Order	1, 33	358117.1<br>9	0.02	<.001	.891	1, 33	0.51	0.14	.003	.713
Reward	1, 33	11705.70	1.20	<.001	.282	1, 33	0.02	0.69	<.001	.412
Order x Reward	1, 33	11705.70	0.72	<.001	.401	1, 33	0.02	1.06	.001	.310
NavonType	1, 33	25480.37	0.64	.001	.431	1, 33	0.03	0.00	<.001	.981
Order x NavonType	1, 33	25480.37	2.66	.004	.112	1, 33	0.03	1.41	.002	.243
Drug	1, 33	41726.04	0.01	<.001	.922	1, 33	0.07	0.00	<.001	.973
Order x Drug	1, 33	41726.04	29.10	.072	<.001	1, 33	0.07	38.96	.103	<.001
Reward x NavonType	1, 33	8298.12	1.20	<.001	.282	1, 33	0.02	0.55	<.001	.462
Order x Reward x NavonType	1, 33	8298.12	0.03	<.001	.857	1, 33	0.02	0.19	<.001	.666
Reward x Drug	1, 33	8082.25	0.63	<.001	.434	1, 33	0.02	0.23	<.001	.631
Order x Reward x Drug	1, 33	8082.25	1.87	<.001	.181	1, 33	0.02	1.13	.001	.296
NavonType x Drug	1, 33	8029.39	0.35	<.001	.557	1, 33	0.02	0.84	<.001	.365
Order x NavonType x Drug	1, 33	8029.39	1.21	<.001	.279	1, 33	0.02	0.26	<.001	.616
Reward x NavonType x Drug	1, 33	15036.61	4.17	.004	0.0245*	1, 33	0.02	3.45	.003	0.036*
Order x Reward x NavonType x Drug	1, 33	15036.61	0.49	<.001	.487	1, 33	0.02	0.06	<.001	.805

* Planned contrast using a directional one-tailed test as per preregistration

Table S7. Repeated measures ANOVAs Error Rate post-goal Navon trials

	ErrorRate					ErrorRate_sqrt				
Effect	df	MSE	F	η²	p	df	MSE	F	η²	p
Order	1, 33	0.04	0.14	.001	.710	1, 33	0.13	0.08	<.001	.778
Reward	1, 33	0.01	3.64	.012	.065	1, 33	0.05	2.58	.009	.118
Order x Reward	1, 33	0.01	0.02	<.001	.899	1, 33	0.05	0.12	<.001	.735
NavonType	1, 33	0.01	0.07	<.001	.788	1, 33	0.04	0.05	<.001	.829
Order x NavonType	1, 33	0.01	0.06	<.001	.811	1, 33	0.04	0.01	<.001	.939
Drug	1, 33	0.02	0.01	<.001	.923	1, 33	0.07	0.01	<.001	.937
Order x Drug	1, 33	0.02	0.40	.002	.533	1, 33	0.07	0.00	<.001	.987
Reward x NavonType	1, 33	0.01	0.34	<.001	.565	1, 33	0.03	1.70	.004	.201
Order x Reward x NavonType	1, 33	0.01	0.05	<.001	.820	1, 33	0.03	0.10	<.001	.756
Reward x Drug	1, 33	0.01	0.16	<.001	.693	1, 33	0.04	0.02	<.001	.887
Order x Reward x Drug	1, 33	0.01	1.27	.003	.268	1, 33	0.04	1.47	.004	.233
NavonType x Drug	1, 33	0.01	1.96	.006	.171	1, 33	0.04	3.66	.010	.064
Order x NavonType x Drug	1, 33	0.01	0.02	<.001	.894	1, 33	0.04	0.05	<.001	.817
Reward x NavonType x Drug	1, 33	0.01	0.59	<.001	0.225*	1, 33	0.02	0.00	<.001	0.4845*
Order x Reward x NavonType x Drug	1, 33	0.01	3.89	.005	.057	1, 33	0.02	2.95	.004	.095

* Planned contrast using a directional one-tailed test as per preregistration

Table S8. Repeated measures ANOVAs RT and RS pre-goal Navon trials

	RT					RS				
Effect	df	MSE	F	η²	p	df	MSE	F	η²	p
Order	1, 33	363423.1<br>5	0.31	.007	.579	1, 33	0.52	0.01	<.001	.924
Reward	1, 33	8918.10	0.93	<.001	.342	1, 33	0.02	0.80	<.001	.377
Order x Reward	1, 33	8918.10	1.42	<.001	.241	1, 33	0.02	2.09	.002	.158
NavonType	1, 33	26674.23	1.86	.003	.182	1, 33	0.05	2.96	.006	.094
Order x NavonType	1, 33	26674.23	0.16	<.001	.694	1, 33	0.05	0.52	.001	.477
Drug	1, 33	71073.57	0.79	.003	.382	1, 33	0.10	0.40	.002	.532
Order x Drug	1, 33	71073.57	18.59	.075	<.001	1, 33	0.10	27.50	.097	<.001
Reward x NavonType	1, 33	7099.22	7.29	.003	.011	1, 33	0.01	4.37	.002	.044
Order x Reward x NavonType	1, 33	7099.22	4.23	.002	.048	1, 33	0.01	2.99	.002	.093
Reward x Drug	1, 33	7377.24	0.01	<.001	.941	1, 33	0.02	0.14	<.001	.711
Order x Reward x Drug	1, 33	7377.24	0.86	<.001	.361	1, 33	0.02	0.39	<.001	.536
NavonType x Drug	1, 33	4331.42	3.23	<.001	.082	1, 33	0.01	4.27	.001	.047
Order x NavonType x Drug	1, 33	4331.42	0.00	<.001	.994	1, 33	0.01	0.00	<.001	.966
Reward x NavonType x Drug	1, 33	7393.36	0.00	<.001	0.4865*	1, 33	0.02	0.03	<.001	0.4325*
Order x Reward x NavonType x Drug	1, 33	7393.36	0.00	<.001	.971	1, 33	0.02	0.23	<.001	.631

* Planned contrast using a directional one-tailed test as per preregistration

Table S9. Repeated measures ANOVAs Error Rate pre-goal Navon trials

	ErrorRate					ErrorRate_sqrt				
Effect	df	MSE	F	η²	p	df	MSE	F	η²	p
Order	1, 33	0.03	0.64	.005	.430	1, 33	0.09	2.22	.015	.146
Reward	1, 33	0.01	1.20	.003	.281	1, 33	0.04	0.29	<.001	.595
Order x Reward	1, 33	0.01	1.34	.004	.255	1, 33	0.04	0.72	.002	.402
NavonType	1, 33	0.02	4.77	.021	.036	1, 33	0.06	2.14	.010	.153
Order x NavonType	1, 33	0.02	0.16	<.001	.695	1, 33	0.06	0.36	.002	.551
Drug	1, 33	0.02	1.24	.005	.273	1, 33	0.05	1.12	.004	.298
Order x Drug	1, 33	0.02	0.14	<.001	.706	1, 33	0.05	0.03	<.001	.870
Reward x NavonType	1, 33	0.01	2.95	.008	.095	1, 33	0.04	2.31	.007	.138
Order x Reward x NavonType	1, 33	0.01	0.02	<.001	.889	1, 33	0.04	0.09	<.001	.762
Reward x Drug	1, 33	0.01	0.94	.002	.339	1, 33	0.03	1.07	.002	.309
Order x Reward x Drug	1, 33	0.01	0.21	<.001	.648	1, 33	0.03	0.77	.002	.386
NavonType x Drug	1, 33	0.01	1.13	.004	.295	1, 33	0.05	1.32	.005	.259
Order x NavonType x Drug	1, 33	0.01	0.00	<.001	.969	1, 33	0.05	0.20	<.001	.654
Reward x NavonType x Drug	1, 33	0.01	0.13	<.001	0.36*	1, 33	0.04	0.00	<.001	0.48*
Order x Reward x NavonType x Drug	1, 33	0.01	0.00	<.001	.985	1, 33	0.04	0.10	<.001	.756

* Planned contrast using a directional one-tailed test as per preregistration

Table S10. Repeated measures ANOVAs RT and RS pre-goal lexical-decision task trials

	RT					RS				
Effect	df	MSE	F	η²	p	df	MSE	F	η²	p
Order	1, 33	258638.6<br>1	0.29	.006	.592	1, 33	0.58	0.16	.004	.690
Reward	1, 33	4843.10	22.08	.009	<.001	1, 33	0.02	21.71	.021	<.001
Order x Reward	1, 33	4843.10	0.38	<.001	.540	1, 33	0.02	0.78	<.001	.385
WordType	1, 33	27172.18	67.61	.134	<.001	1, 33	0.03	238.50	.222	<.001
Order x WordType	1, 33	27172.18	0.24	<.001	.626	1, 33	0.03	0.27	<.001	.608
Drug	1, 33	53170.71	0.37	.002	.545	1, 33	0.07	0.84	.003	.367
Order x Drug	1, 33	53170.71	18.28	.076	<.001	1, 33	0.07	38.03	.105	<.001
Reward x WordType	1, 33	1460.92	0.60	<.001	.445	1, 33	0.01	2.76	<.001	.106
Order x Reward x WordType	1, 33	1460.92	0.95	<.001	.336	1, 33	0.01	0.12	<.001	.727
Reward x Drug	1, 33	2970.41	6.05	.002	0.0095*	1, 33	0.01	1.79	<.001	0.095*
Order x Reward x Drug	1, 33	2970.41	0.20	<.001	.656	1, 33	0.01	1.55	<.001	.221
WordType x Drug	1, 33	10930.20	0.13	<.001	.718	1, 33	0.01	0.02	<.001	.886
Order x WordType x Drug	1, 33	10930.20	9.47	.009	.004	1, 33	0.01	3.05	<.001	.090
Reward x WordType x Drug	1, 33	1306.25	0.12	<.001	.735	1, 33	0.00	0.97	<.001	.332
Order x Reward x WordType x Drug	1, 33	1306.25	0.01	<.001	.944	1, 33	0.00	0.17	<.001	.682

* Planned contrast using a directional one-tailed test as per preregistration

Table S11. Repeated measures ANOVAs Error Rate pre-goal lexical-decision task trials

	ErrorRate					ErrorRate_sqrt				
Effect	df	MSE	F	η²	p	df	MSE	F	η²	p
Order	1, 33	0.02	0.01	<.001	.918	1, 33	0.06	0.00	<.001	.995
Reward	1, 33	0.00	5.28	.010	.028	1, 33	0.02	7.59	.021	.009
Order x Reward	1, 33	0.00	3.47	.007	.072	1, 33	0.02	1.59	.004	.216
WordType	1, 33	0.02	22.53	.156	<.001	1, 33	0.05	23.87	.153	<.001
Order x WordType	1, 33	0.02	0.67	.005	.419	1, 33	0.05	1.43	.011	.240
Drug	1, 33	0.01	0.10	<.001	.759	1, 33	0.02	0.00	<.001	.958
Order x Drug	1, 33	0.01	0.51	.002	.480	1, 33	0.02	1.37	.005	.249
Reward x WordType	1, 33	0.00	0.00	<.001	.997	1, 33	0.01	0.88	.002	.355
Order x Reward x WordType	1, 33	0.00	0.03	<.001	.870	1, 33	0.01	0.01	<.001	.937
Reward x Drug	1, 33	0.00	0.43	<.001	0.259*	1, 33	0.01	0.31	<.001	0.29*
Order x Reward x Drug	1, 33	0.00	1.03	.001	.317	1, 33	0.01	0.18	<.001	.677
WordType x Drug	1, 33	0.00	0.19	<.001	.666	1, 33	0.01	0.77	.002	.385
Order x WordType x Drug	1, 33	0.00	2.69	.006	.110	1, 33	0.01	7.43	.015	.010
Reward x WordType x Drug	1, 33	0.00	3.72	.005	.062	1, 33	0.01	1.89	.003	.179
Order x Reward x WordType x Drug	1, 33	0.00	2.33	.003	.137	1, 33	0.01	1.78	.003	.191

* Planned contrast using a directional one-tailed test as per preregistration

Figure S1. Distribution of residuals before and after transformation

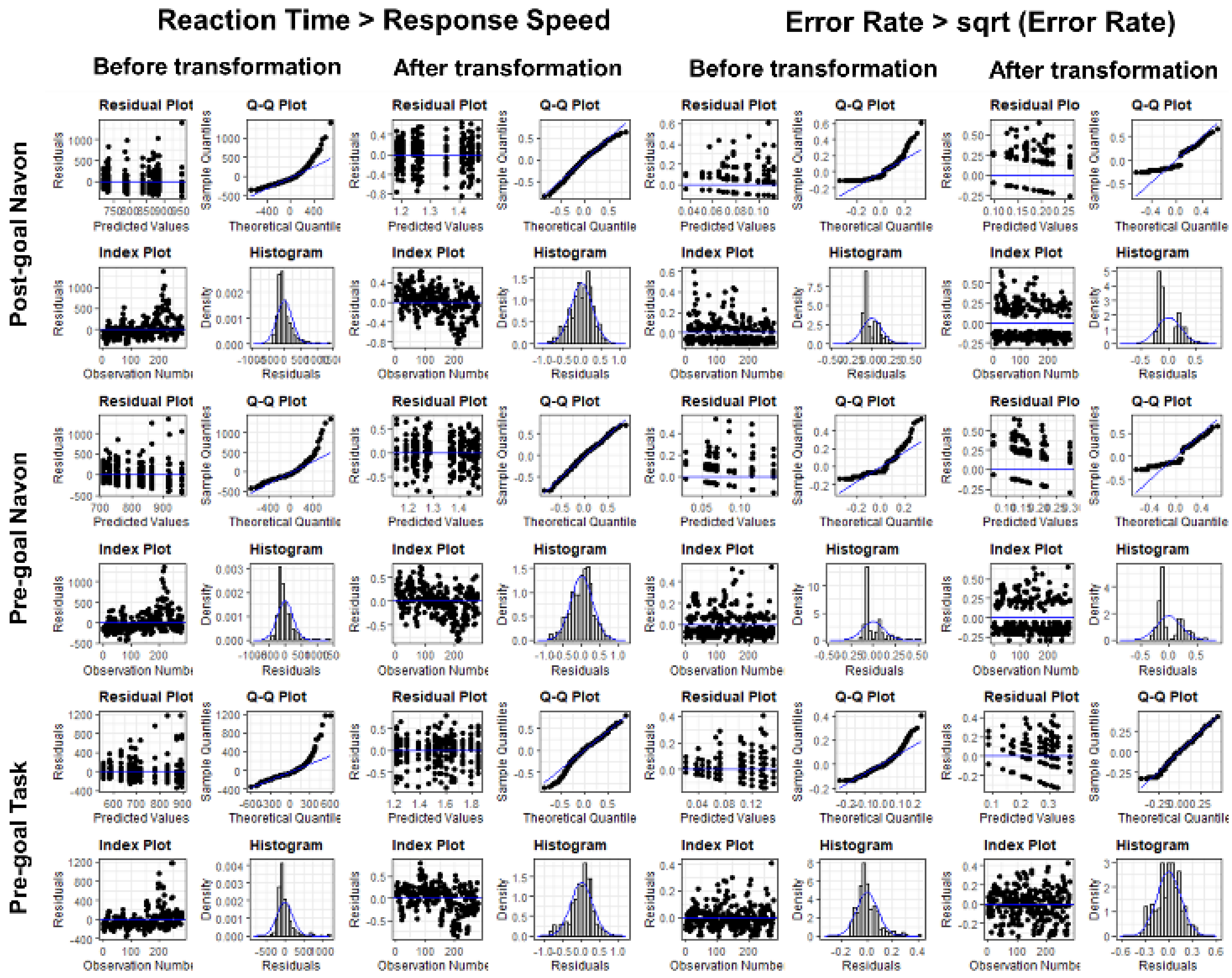

Figure S2.

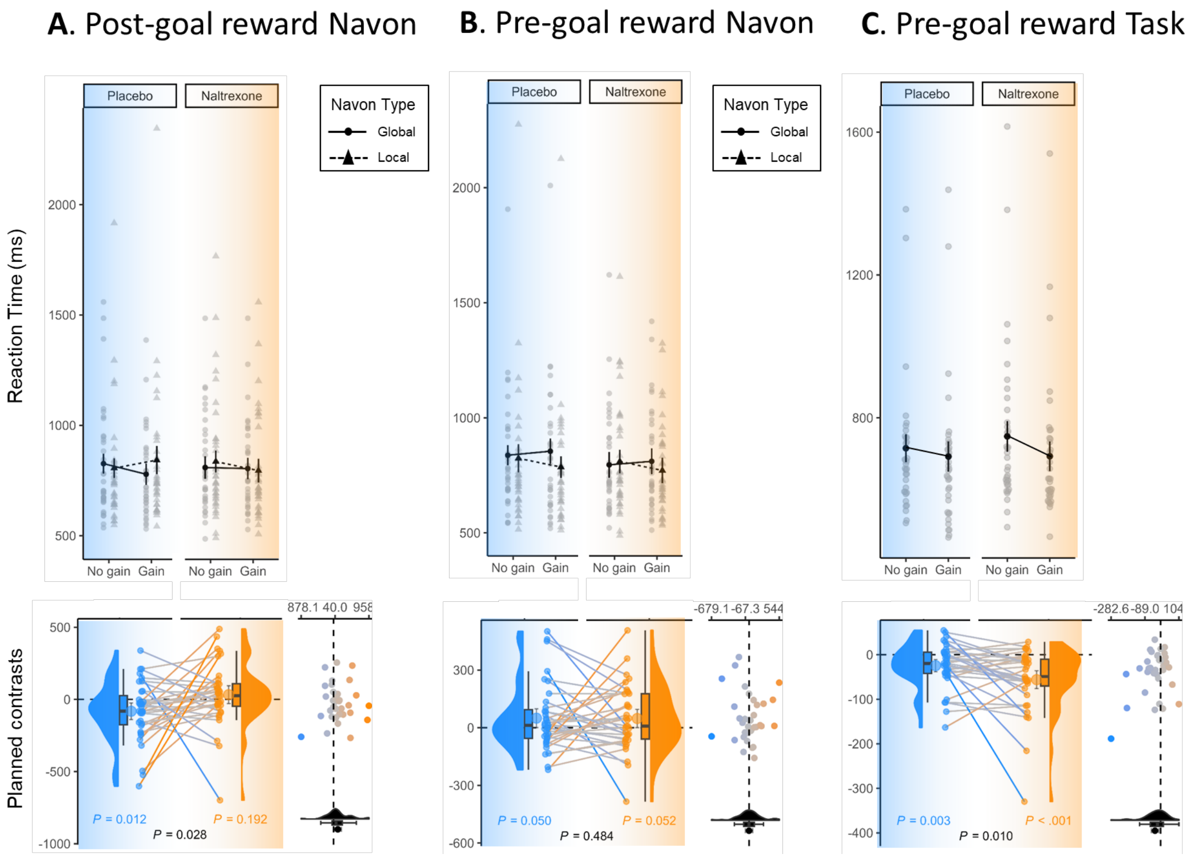

Opioid blockade (Naltrexone condition; orange) relative to placebo (blue) selectively eliminated attentional broadening after reward receipt (A), but did not change attentional narrowing after reward anticipation (B). The drug appeared to influence reward-related performance speed on the lexical decision task in a direction opposite to the predictions (C). Upper panels show the mean and 95% within-subject confidence intervals, and dots indicate data from each participant. Lower panels show the values of the planned contrast of interest to test the interaction between Reward and Navon Type (A and B; difference of difference scores) and the main effect of Reward (C; difference score) against zero (dotted horizontal line) for the placebo (blue) and naltrexone (orange) condition separately. The paired difference between these contrasts (i.e., the slope of the lines connecting the dots) are plotted in the scatterplot (x-axis) as a function of the average of both values (y-axis) and were also tested against zero (dotted vertical line). The error bars show the mean and 90% within-subject confidence intervals, and reported p-values are one-tailed and use t-tests against zero that ignore all other factors of the design.
